# Optical Coherence Tomography as a Biomarker for Diagnosis, Progression, and Prognosis of Neurodegenerative Diseases

**DOI:** 10.1155/2016/8503859

**Published:** 2016-10-20

**Authors:** Maria Satue, Javier Obis, Maria J. Rodrigo, Sofia Otin, Maria I. Fuertes, Elisa Vilades, Hector Gracia, Jose R. Ara, Raquel Alarcia, Vicente Polo, Jose M. Larrosa, Luis E. Pablo, Elena Garcia-Martin

**Affiliations:** ^1^Ophthalmology Department, Miguel Servet University Hospital, Zaragoza, Spain; ^2^IIS Aragon, Institute for Health Sciences of Aragon (IACS), Zaragoza, Spain; ^3^Neurology Department, Miguel Servet University Hospital, Zaragoza, Spain

## Abstract

Neurodegenerative diseases present a current challenge for accurate diagnosis and for providing precise prognostic information. Developing imaging biomarkers for multiple sclerosis (MS), Parkinson disease (PD), and Alzheimer's disease (AD) will improve the clinical management of these patients and may be useful for monitoring treatment effectiveness. Recent research using optical coherence tomography (OCT) has demonstrated that parameters provided by this technology may be used as potential biomarkers for MS, PD, and AD. Retinal thinning has been observed in these patients and new segmentation software for the analysis of the different retinal layers may provide accurate information on disease progression and prognosis. In this review we analyze the application of retinal evaluation using OCT technology to provide better understanding of the possible role of the retinal layers thickness as biomarker for the detection of these neurodegenerative pathologies. Current OCT analysis of the retinal nerve fiber layer and, specially, the ganglion cell layer thickness may be considered as a good biomarker for disease diagnosis, severity, and progression.

## 1. Introduction

Neurodegenerative disorders present a current challenge for accurate diagnosis and for providing precise prognostic information. Some diseases, such as multiple sclerosis (MS), present with an unpredictable course, whereas others, such as Parkinson disease (PD) and Alzheimer's disease (AD), may take several years to obtain a definitive diagnosis. Due to increased aging population in developed countries, neurodegenerative diseases such as PD and AD have become more prevalent and thus new technologies and more accurate tests are needed to improve and accelerate the diagnostic procedure in early stages of these diseases.

Developing imaging biomarkers for MS, PD, and AD in order to provide early diagnosis and predict the clinical course and future disability will improve the clinical management of these patients and may be useful for monitoring treatment effectiveness as well.

Optical coherence tomography (OCT) provides cross-sectional imaging of internal structures in biological tissues [1, 2]. Recent research using OCT technology has demonstrated that parameters provided by OCT are accurate to detect various inner retinal or optic nerve pathologies. In the last decade this technique has also been applied in several areas in neurology, demonstrating its potential role as a fundamental tool in the study of neurodegenerative diseases, such as MS, PD, or AD [3–9]. At the present time, however, no clear guidelines are available on whether one, several, or all of the retinal parameters measured by OCT can be used in the diagnosis of these pathologies, and therefore the use of OCT technology in the clinical management of neurological patients is limited to follow disease progression in several common pathologies.

In the present article, we review the application of retinal evaluation using OCT technology to provide better understanding of the possible role of the retinal layers thickness as biomarker for the detection of neurodegenerative pathologies such as MS, PD, and AD.

## 2. Multiple Sclerosis

Multiple sclerosis (MS) is a neurodegenerative disease characterized by demyelination and axonal degeneration in the central nervous system, leading to progressive neurologic deficits [10, 11]. Axonal damage already occurs in the early stages of the disease, not being related to inflammatory or autoimmune episodes against myelin [12, 13].

The retina of patients with MS displays inflammatory and neurodegenerative findings, such as perivascular inflammatory infiltrates and atrophy of the inner retinal layers [14]. In 1999, Parisi et al. reported for the first time a significant reduction in the retinal nerve fiber layer (RNFL) of patients with MS and previous optic neuritis (MSON) compared to healthy subjects and its correlation with pattern electroretinogram changes in these eyes [15]. Since then, an increased interest in the application of OCT technology led to a large number of studies on the retinal changes in MS patients with and without previous ON episodes (non-ON). So far, studies using spectral-domain OCT have revealed that the retina in non-ON eyes shows thinner peripapillary RNFL (pRNFL) than healthy controls [16–19].

### 2.1. RNFL Thickness as a Biomarker of Disease Severity and Progression in MS

The introduction in the last few decades of OCT in the study of MS has provided new information on correlations between visual deficiencies and retinal alterations in these patients and also between pRNFL thinning and disability [9]. Recent studies using OCT showed that low contrast letter acuity scores in MS patients reflect the axonal and neuronal losses in the anterior visual pathway (observed as RNFL and retinal neuronal layer thinning quantified using OCT technology) [20, 21]. Saidha et al. [20] demonstrated the presence of retinal ganglion layer thinning in patients with relapsing-remitting and progressive MS and its correlation with high and low contrast visual acuity scores. Similarly, Burkholder et al. showed a significant correlation between altered visual function scores and reduced macular volume in these patients [21]. More importantly, retinal measures in MS patients evaluated using OCT technology seem to correlate directly with brain-substructure volumes and grey and white matter volumes and inversely with FLAIR-lesion volume, as objectified by MRI, thus reflecting a possible correlation with general central nervous system pathology in MS [22].

This axonal loss in MS, as observed by OCT, is associated with physical and cognitive disability as measured by the Expanded Disability Status Scale (EDSS) [23–25] and has demonstrated its utility as a biomarker of disease progression [5, 22, 26, 27]. Recently, pRNFL atrophy was associated with worsening disability and lower quality of life [28]. Garcia-Martin et al. analyzed the structural change in the retina of MS patients for a time lapse of 3 years and demonstrated that a reduction in the pRNFL thickness in these patients was associated with lower quality of life (measured using the MSQOL-54 questionnaire) and greater disability. The MSQOL-54 questionnaire is a multidimensional health-related quality of life measure that combines both generic and MS-specific items into a single instrument. This 54-item questionnaire generates 12 subscales (physical function, role limitations: physical, role limitations: emotional, pain, emotional well-being, energy, health perceptions, social function, cognitive function, health distress, overall quality of life, and sexual function) along with two summary scores (the physical health composite summary and the mental health composite) and two additional single-item measures (satisfaction with sexual function and change in health). In Garcia-Martin's study the physical health composite of the MSQOL-54 questionnaire (composed of different questions about the patient's perception of their physical condition to fulfill every day's tasks) was especially correlated with pRNFL thickness. Additionally, the baseline mean and superior pRNFL thicknesses appear to predict decreases in the quality of life in patients with MS [28].

**Figure 1:**
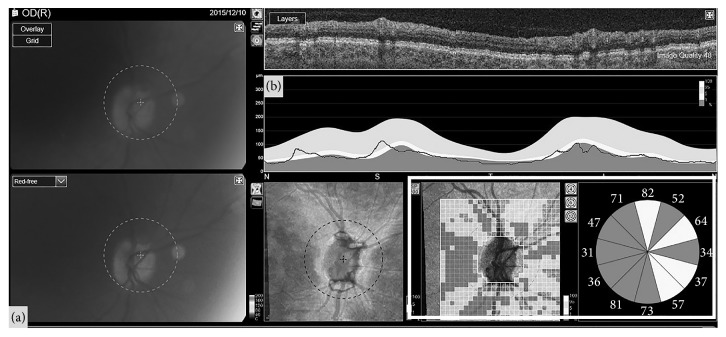
Optic nerve head (a) and retinal nerve fiber layer analysis (b) as obtained with swept-source optical coherence tomography in a 43-year-old patient with multiple sclerosis who suffered a previous episode of optic neuritis 5 years ago. The pixel map and the clock sector analysis (marked with the white square) of the optic disc shows important retinal nerve fiber layer loss in most sectors of the peripapillary area.

**Figure 2:**
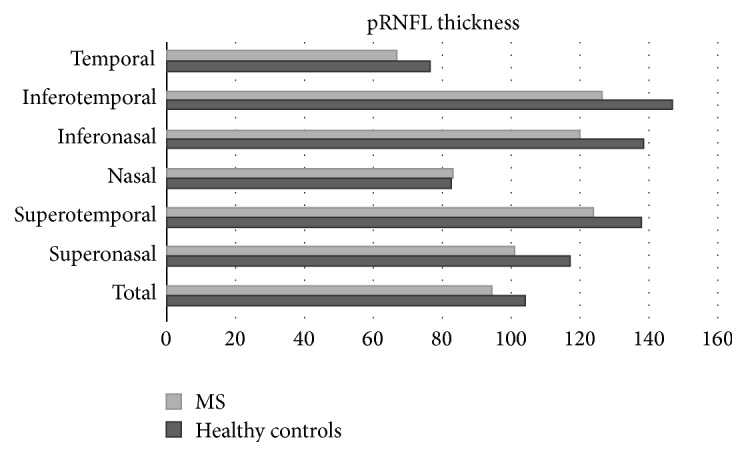
Mean peripapillary retinal nerve fiber layer (pRNFL) thickness of 100 multiple sclerosis (MS) patients compared with 97 healthy controls, as measured with optical coherence tomography. The peripapillary area is divided into 6 different sectors (superonasal, superotemporal, nasal, inferonasal, inferotemporal, and temporal) and average thickness. All measurements except nasal thickness were found to be significantly reduced in MS patients compared to controls (Garcia-Martin et al., data not published).

RNFL thickness decreases with normal aging [29]. However, compared to healthy subjects, MS patients present with a higher reduction and more affected sectors of the pRNFL thickness (Figures 1 and 2) and this reduction seems to be even greater in untreated patients [30]. More recently, the pRNFL thickness was pointed out as a good predictor of the likelihood of disability worsening in MS patients over time [9]. Patients who had a pRNFL ≤ 92-93 *μ*m showed a 58% increase in the risk of disability worsening, and patients in the lower pRNFL thickness tertile displayed increased risk of disability worsening compared to those in the higher tertile. Patients with pRNFL ≤ 87/88 *μ*m doubled the risk of disability worsening at any time after the first year and until the third year of follow-up. This disability worsening prediction by pRNFL seems to be dependent on the follow-up time, since this risk almost increased fourfold after the third year and until the fifth year of follow-up. This increased risk of disability worsening was present in patients with and without a previous ON episode, although it was higher in patients with MSON [9].

Combined RNFL parameters were also demonstrated to improve the ability of this technology to distinguish between eyes from MS patients and eyes from healthy subjects, by calculating a linear discriminant function [6]. Mathematical analysis showed that a linear discriminant function where different RNFL parameters (thickness in different sectors) were combined yielded the highest sensitivity at a high specificity compared to any single sector of the OCT parameters [6].

### 2.2. Retinal Segmentation Analysis: Ganglion Cell Layer Thickness as a Biomarker for MS

Histopathologic evaluation of postmortem MS eyes revealed the loss of inner nuclear layer neurons and significant ganglion cell and inner plexiform layer (GCIPL) atrophy [14], even in cases where the number of axons remained intact [31]. New spectral-domain (SD) OCT segmentation software allows for the measurement of the various retinal layers separately, taking in vivo measurements one step closer to histologic observations. Current studies using this segmentation analysis software demonstrated a reduction of the inner retinal layers, including the GCIPL, suggesting ganglion cell loss [20, 32–34]. Moreover, this GCIPL reduction is also correlated with reduced visual function, functional disability as measured by the EDSS, and vision-specific quality of life in MS patients [32, 34]. Does this all mean that the GCIPL could be a more precise biomarker than the RNFL? In a recent study comparing both GCIPL and pRNFL thickness, average GCIPL was altered more frequently than average pRNFL, and GCIPL thickness was demonstrated to have better sensitivity than temporal pRNFL thickness for detecting retinal thickness changes in patients with MS [35, 36]. Additionally, logistic regression analysis demonstrated that GCIPL thickness is a potential predictor of axonal damage in patients with MS, whereas the thickness of all other retinal layers (including the RNFL) was not predictive of axonal atrophy [34]. Thus, GCIPL thickness has rapidly emerged as a useful structural biomarker in MS, even better than RNFL thickness, probably because the neuronal cell bodies suffer an earlier affectation than the retinal axons in MS. Some authors, ophthalmologists and neurologists, have suggested that OCT measurements may be more accurate than MRI parameters to determine progression in MS patients [9, 34].

## 3. Parkinson Disease

Parkinson's disease (PD) is the second most common neurodegenerative disorder in the developed world (after Alzheimer's disease) and is characterized by motor symptoms, such as resting tremor, bradykinesia, and rigidity. However, a large variety of nonmotor symptoms are also present in this disease: mood [37, 38], cognitive dysfunction [39], autonomic failure [40], and sleep disorders [41] are highly common in PD patients.

Vision is one of the nonmotor systems altered in PD, reporting decreased visual acuity, contrast sensitivity, or colour vision reduction [42–48].

Postmortem neurochemical analysis of eyes of deceased patients diagnosed with PD has shown decreased retinal dopamine concentration [49, 50]. Dopamine in the human retina is released by a set of amacrine cells located in the proximal inner nuclear layer of the retina. These dopaminergic cells communicate with other types of amacrine cells modulating the interconnections between bipolar and retinal ganglion cells and also send long processes to other retinal layers, thus playing a pivotal role in channelling visual information “vertically” through the retina [51]. Dopamine in the mammalian retina modulates colour vision and contrast sensitivity through dopaminergic receptors (D1 and D2), which are differentially located in the retinal layers. A complete lack of this dopaminergic receptor activation leads to signal dispersion and alterations in colour vision and contrast sensitivity.

### 3.1. RNFL Thickness as a Possible Biomarker for PD Diagnosis

Retinal changes in PD were first reported in 2004 by Inzelberg et al. [52], who demonstrated RNFL thickness reduction in the peripapillary area in a small group of 10 PD patients. In the following years, research on this topic increased dramatically and Inzelberg's results were confirmed by other studies using time-domain OCT [53–55]. Mean and temporal pRNFL thickness seem to be most affected based on these studies. Later, studies using SD-OCT also demonstrated significant reduction of the inferior pRNFL thickness (along with mean and temporal reduction) [56]. Retinal thickness in the macular area and total macular volume are also significantly reduced in PD [53, 56–58]; however not all studies demonstrating macular thinning in these patients could find similar differences in the pRNFL measurements [59–61].

### 3.2. Macular Thickness as a Biomarker for Disease Progression and Severity

Macular measurements appear to be an important feature in PD. Based on Spectralis OCT measurements, a linear discriminant function was designed by Garcia-Martin et al. to combine parameters improving the diagnostic ability of OCT: a calculated retinal linear discriminant function including different macular thickness measurements yielded the highest sensitivity at a high specificity compared to any single parameter determined using OCT or any other linear discriminant function calculated from pRNFL measurements, suggesting that macular measurements in PD could be a stronger marker for PD diagnosis [8]. A remodelling of the foveal pit caused by PD has also been suggested [62].

There is an association between macular thinning and disease progression and severity in PD. Altintaş et al. demonstrated a relation between PD severity and alterations in foveal thickness using time-domain OCT [54]. An association between disease severity as measured by the Hoehn Yahr scale and macular thickness was also found using SD-OCT [57, 63]. Disease severity based on the commonly used Unified Parkinson's Disease Rating Scale III (UPDRS III) and quality of life (based on the Schwab England scale) is also correlated with macular measurements, especially temporal and inferior sectors [57, 63]. Contrary to macular measurements, correlations between disease severity/duration and pRNFL thickness have proved to be scarce or nonexistent [55, 57].

### 3.3. Retinal Segmentation Analysis: Ganglion Cell Layer Thickness as a Biomarker for PD

**Figure 3:**
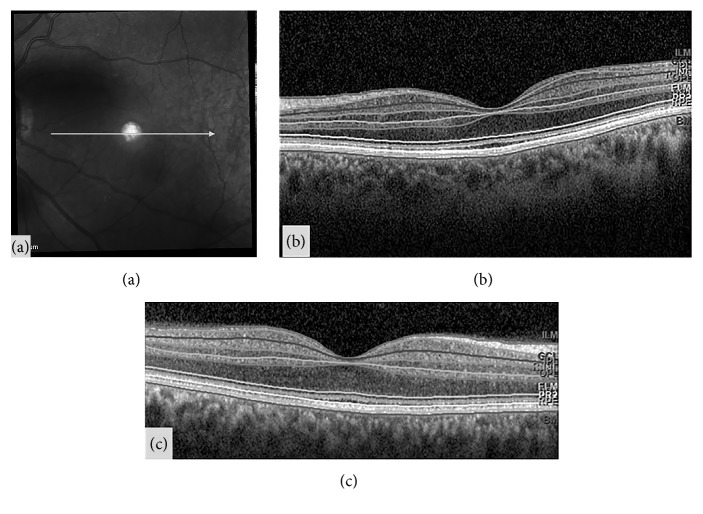
Example of segmentation analysis of the different retinal layers, in a cross-sectional linear scan of the macular area (a), obtained with Spectralis optical coherence tomography, in a healthy control (b) and a patient diagnosed with Parkinson disease (c). The marked lines are automatically provided by the segmentation software and represent the different layers of the retina. Corresponding acronyms are also provided by the segmentation software: ILM: inner limiting membrane; GCL: ganglion cell layer; IPL: inner plexiform layer; INL: inner nuclear layer; OPL: outer plexiform layer; ONL: outer nuclear layer; ELM: external limiting membrane; PR: photoreceptors; MB: Bruch's membrane.

**Figure 4:**
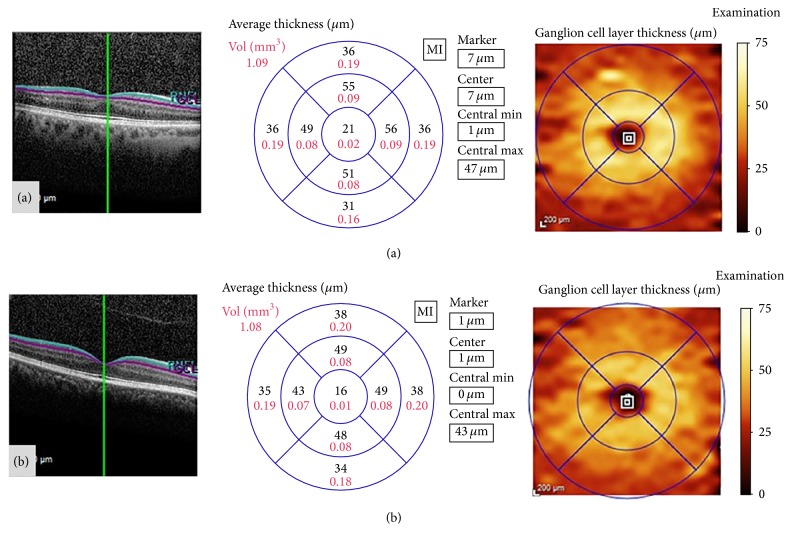
Example of segmentation analysis of the macular ganglion cell layer, obtained with Spectralis optical coherence tomography, in a healthy control (a) and a patient diagnosed with Parkinson disease (b). The segmentation report shows the ganglion cell layer thickness (in microns) and total volume (in mm^3^) of the ETDRS macular area. In this patient (b), the central and inner macular areas present thinning of the ganglion cell layer, compared with the healthy control (a).

As research on PD moved forward, SD-OCT segmentation analysis was also applied to the evaluation of the retina of PD patients (Figures 3 and 4). In a recent study by Blennow et al., the inner retinal layer (IRL, defined as the internal limiting membrane + nerve fiber layer + ganglion cell layer + inner plexiform layer down to the inner nuclear layer interface) was found to be reduced in the perifoveal area of PD patients compared to healthy subjects [64]. Furthermore, when single retinal layers were measured, reductions in the macular RNFL, the ganglion cell layer (GCL), the inner plexiform layer (IPL), the inner nuclear layer, and the outer plexiform layer were demonstrated [63]. However, only the GCL predicts axonal damage in PD patients [63]. Segmentation analysis also revealed that the inner retinal layers of the macular area (RNFL, GCL, and IPL) are more affected with disease duration and that GCL thickness is inversely correlated with disease duration and disease severity [63]. Therefore and based on these recent segmentation studies the inner retinal layers of the macular area should be pointed at as the strongest biomarkers for PD diagnosis and progression.

## 4. Alzheimer's Disease

Alzheimer's disease (AD) is the most frequent cause of dementia worldwide [64]. Although it is most commonly associated with memory deficits and cognitive impairment, patients with AD also exhibit alterations in visual processing [65–67]. Colour vision and contrast sensitivity alterations are frequently present and have been suggested as predictors for cognitive dysfunction [66].

### 4.1. RNFL Measurements in Alzheimer's Disease

It has been postulated that defects in the pRNFL may be the earliest sign of AD, even before damage to the hippocampus occurs [68]. A reduction in the pRNFL thickness was observed in AD patients [69–71], especially in mean and inferior sectors [72, 73]. However, one study did not find significant differences in the pRNFL thickness between AD patients and healthy controls [74].

### 4.2. Macular Measurements as a Biomarker of Disease Severity in AD

Macular thickness and macular volume are importantly affected in patients with AD [72–75] and a correlation between macular volume and cognitive impairment was suggested [71]. Although foveal thickness is not considered a useful parameter to detect atrophy in AD [76], the inner and outer ETDRS sectors of the macula seem to be highly affected in these patients [73]. However, it is the combination of the pRNFL parameters (in a calculated linear discriminant function) that seems to show the highest diagnostic accuracy in AD, compared to combined macular thickness measurements or single thickness sectors [73]. More studies on discriminant linear function including macular volume are needed to corroborate whether macular measurements are a good biomarker for AD diagnosis.

### 4.3. Retinal Segmentation Analysis: Ganglion Cell Layer Thickness as a Biomarker for Diagnosis and Disease Severity in AD

Taking a step further into research on retinal biomarkers for AD, segmentation analysis of the retinal layers was recently introduced in AD studies. A previous study on postmortem AD patients did not find any evidence for ganglion cell loss compared to controls [77]. However, the sample size in Curcio study was extremely small. Other histopathological studies have suggested that disease pathology in the precortical visual system (i.e., the retina and optic nerve) is a possible mechanism underlying visual impairments observed in AD patients and may be related to ganglion cell alterations. Different sets of ganglion cells (parvocellular, magnocellular, and koniocellular ganglion cells) located in the retinal GCL result in three different pathways which identify colour and spatial contrast at different frequencies [78–80]. Previous studies (histologic, electroretinogram, and imaging studies) of these pathways suggest that the general loss of magnocellular and parvocellular cells of the retina is likely to be an important contributory mechanism for visual impairment in AD [81]. Dendritic atrophy and loss of retinal ganglion cells have also been observed in the retina of a mouse model of AD, where the accumulation of beta-amyloid in the inner retinal layers was observed [82]. These beta-amyloid deposits may be responsible for the depletion of parvo- and magnocellular cells in the retina and may be linked to visual function impairment. Moreover, results of this study suggest that dendritic atrophy of the retinal ganglion cells precedes ganglion cell loss. Since dendrites of the ganglion cells are confined to the IPL, this layer could also play a major role as a biomarker for neuronal damage in AD [82].

According to previous studies, recent animal research showed inner retinal dysfunction in a mouse model for AD [83]. Retinal segmentation analysis with OCT in this animal model demonstrated RNFL thinning, but no associated changes were observed in the ganglion cell complex [83]. Contrary to the animal model, patients with AD present a reduction of the RNFL, GCL, and IPL observable with the OCT segmentation software [84, 85], and these inner layers are also more affected in those patients with longer course of the disease [85]. Importantly, when compared to RNFL thickness, GCIPL presents higher sensitivity to discriminate AD patients from controls [84]. Moreover, the GCL and IPL are predictors of axonal damage in these patients and GCL is associated with disease duration and severity [85]. Based on these findings, it is possible that the combination of measurements of the retinal inner layers might be the ultimate biomarker for diagnosis and progression in AD.

## 5. Future Directions

The unique accessibility of the retina and optic nerve to in vivo measurements and the structure-function correlations provided by the afferent visual system in multiple sclerosis, Parkinson's disease, and Alzheimer's disease make the analysis of the retinal structures a useful model system to test new therapies. However, there are currently very few studies focusing on the evaluation of treatment effectiveness through OCT analysis. Further research remains to be done in a number of areas, including practical aspects of implementing clinical outcome measures in multicentre studies, further validation of other biomarkers (fluid-based biomarkers and other imaging techniques) development, and the evaluation of new different therapies effectiveness. Longitudinal studies are also key in the development of biomarkers for disease progression. Most studies evaluated in this review include only cross-sectional data, which is an important limitation for the analysis of imaging biomarkers, especially for disease progression and treatment effectiveness. We believe more longitudinal studies should be carried out, especially in PD and AD patients, since progressive changes in these two diseases have not yet been investigated.

## 6. Conclusions

In the past decade, OCT technology has proved its utility in the diagnosis and progression of neurodegenerative diseases. Numerous clinical studies have demonstrated that the RNFL and macular thickness are useful markers for disease progression and prognosis in MS, PD, and AD. New OCT segmentation software has also allowed better understanding of the physiopathology of axonal degeneration in these neurological diseases through the objective observation of the different retinal layers. Recent research using the latest imaging technology in ophthalmology has demonstrated that an early damage of the anterior visual pathway occurs in MS, PD, and AD and that the ganglion cell layer is the ultimate biomarker for disease diagnosis, severity, and progression. Thus, OCT technology should be used as a common and very useful clinical complement in the diagnosis and control of neurodegenerative disorders.
